# Taller-than-wide as a red flag for malignancy in ultrasound of parotid gland tumors

**DOI:** 10.1007/s00405-025-09832-9

**Published:** 2025-11-12

**Authors:** Andreas Spörlein, Valentin Burkhardt, Tobias Schulz, Naglaa Mansour, Kathrin Gerstacker, Andreas Knopf

**Affiliations:** Department of Otorhinolaryngology – Head and Neck Surgery, Medical Center University of Freiburg, Freiburg, Germany

**Keywords:** Parotid gland tumors, Taller-than-wide sign, Ultrasound risk stratification, Multiparametric scoring system, Head and neck ultrasound

## Abstract

**Purpose:**

Preoperative evaluation of malignancy in parotid gland tumors is challenging due to heterogeneous and non-specific tissue characteristics. The taller-than-wide (TTW) criterion, validated in thyroid and breast tumors, may also be relevant for parotid gland tumors, as malignant growths typically invade beyond microanatomic boundaries.

**Materials and methods:**

In this retrospective study, 140 parotid neoplasms were evaluated via preoperative ultrasound and definitive histopathology. TTW was defined as the dimension perpendicular to the skin exceeding the largest parallel dimension. TTW was incorporated into a previously validated risk score, and additional clinical and sonographic features associated with malignancy were identified.

**Results:**

Malignant tumors more frequently exhibited TTW than benign tumors (27.3% vs. 3.4%, *p* < 0.01). The mean TTW ratio was higher in malignant lesions (0.81 ± 0.29) compared with benign lesions (0.67 ± 0.17, *p* < 0.01). Using a TTW ratio cutoff of 1 yielded high specificity (96.6%) but low sensitivity (27.3%). A multiparametric risk score including TTW demonstrated excellent discriminatory ability (AUC 0.89) with a sensitivity of 81.2% and specificity of 89.0%.

**Conclusion:**

TTW should be considered an adjunctive red flag for malignancy in parotid gland tumors but lacks sensitivity for standalone use. A composite risk score integrating TTW and other readily assessable parameters offers robust preoperative risk stratification, potentially guiding the extent of surgical intervention.

**Supplementary Information:**

The online version contains supplementary material available at 10.1007/s00405-025-09832-9.

## Introduction

Salivary gland neoplasms comprise a heterogeneous group of benign and malignant tumors, with the majority arising in the parotid gland (70%) but showing markedly different behaviors, prognoses, and treatment strategies [1, 2]. Although malignant tumors of the parotid gland are relatively rare, accounting for 3–5% of all head and neck malignancies, they present significant clinical challenges because of their diverse histopathological features and potential for local invasion, perineural spread, and distant metastases [2–4].

Ultrasound plays a pivotal role in the initial evaluation of parotid gland lesions, given its widespread availability, excellent soft-tissue resolution, and capacity for real-time assessment of vascularity via color Doppler imaging [5–7]. Ill-defined margins are typical sonographic findings suggestive of malignancy and other features including heterogeneity of echotexture, increased intralesional vascularity, and elevated tissue stiffness on elastography have been discussed [8–10]. However, the wide histopathological heterogeneity and overlapping imaging features can make it difficult to distinguish benign from malignant parotid tumors preoperatively [11, 12]. A risk score for identifying malignant salivary gland tumors has been proposed, but has not been externally validated yet [9, 13].

Preoperative risk assessment is crucial for adequate surgical planning of the resection [14]. An extracapsular dissection or partial parotidectomy is sufficient for most benign lesions [15, 16]. A total or even radical parotidectomy, with or without neck dissection, is required for many malignant parotid lesions with the exception of superficially located T1 or T2 low-grade tumors for which extracapsular dissection can be considered in the absence of adverse pathological features [16, 17].

In thyroid ultrasound, the taller-than-wide (TTW) shape is widely recognized as an indicator of malignancy, reflecting neoplastic growth perpendicular to normal tissue planes. TTW nodules are consistently placed into higher risk categories in major stratification systems, including ACR TI-RADS [18–20]. In breast ultrasound, a “non-parallel” (or TTW) orientation is included in the BI-RADS lexicon and has been shown to raise suspicion for malignancy [21]. Consequently, TTW has become a valuable ultrasonic criterion across different anatomic sites, offering a straightforward measure of malignant potential.

If proven reliable for parotid lesions, TTW could serve as a quantitative adjunct to existing ultrasound criteria, thereby improving diagnostic accuracy. Moreover, integrating TTW measurements with other established features might improve existing sonographic risk stratification systems [9]. The integration of multiple, easily measurable ultrasound features could also be employed for algorithmic tumor evaluation through machine learning methods [22, 23].

The present study aims to investigate the diagnostic value of the TTW criterion, among other factors, in differentiating benign and malignant parotid gland tumors. By systematically correlating this quantitative sonographic parameter with definitive histopathological diagnoses, the study seeks to contribute to ultrasound-based risk assessment of parotid tumors.

## Materials and methods

Patients who consecutively underwent partial or total parotidectomy for a parotid gland tumor at a large tertiary university hospital in 2023 were retrospectively analyzed. Those who underwent parotidectomy for inflammation or fistula, for achieving adequate resection margins in skin cancer, or those with incomplete documentation were excluded. A routine, structured preoperative ultrasound examination was performed using a linear transducer (PLT-1005BT, 10 MHz, Aplio i800 and Aplio a, Canon Medical Systems, Ōtawara, Japan). This ultrasound protocol included evaluation of the affected parotid gland, the other major salivary glands including the contralateral parotid, cervical lymph node status, and the thyroid gland. Images of the parotid gland masses were acquired in two planes. Color Doppler sonography was used to visualize vascularization in most examinations. Following resection, a histopathological analysis was performed. Patient data, including age, sex, smoking history, symptoms, and histopathological parameters were collected.

**Fig. 1:**
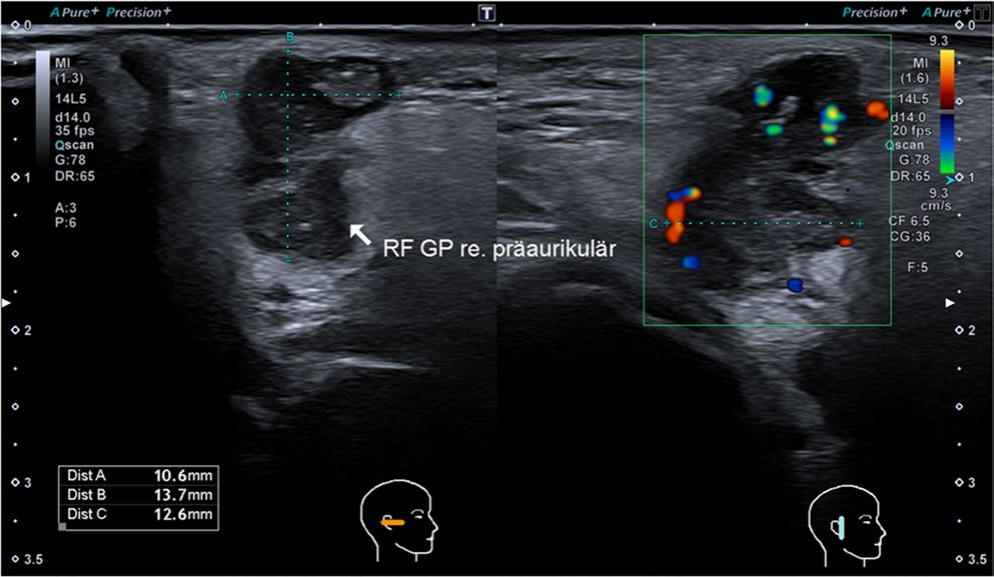
Sonographic image of a parotid gland tumor meeting the taller-than-wide (TTW) criterion, with a TTW ratio of 1.09. Histopathologic analysis revealed the diagnosis of a metastasis of a squamous cell carcinoma of the skin. Dist A: anterior-posterior dimension, Dist B: orthogonal dimension, Dist C: craniocaudal dimension

The ultrasound images were evaluated by a blinded investigator. The anterior-posterior dimension, craniocaudal dimension, and the dimension orthogonal to the skin were measured. The TTW criterion was met if the orthogonal dimension exceeded both the anterior-posterior and craniocaudal dimensions (Fig. 1). A ratio of the extension orthogonal to the skin divided by the largest extension parallel to the skin was formed (TTW ratio). The form of the tumor was assessed for being irregular or spiculated as opposed to round, oval or lobular. The border of the tumor was assessed and rated as either smooth or not smooth. The internal echo of the tumor was classified as either homogenous or non-homogenous, and the echogenicity was classified as anechoic, hypoechoic, isoechoic or hyperechoic in comparison to the surrounding salivary tissue. In case of non-homogenous internal echo, only the predominant echogenicity was considered. The presence of distal acoustic enhancement, calcification, and skin invasion was noted. Vascularization, as evaluated by color Doppler sonography, was categorized as avascular, peripheral, central, or ubiquitous; vascularization intensity was classified visually as avascular, moderate, or strong.

Statistical analysis was performed in GraphPad Prism (Version 10.4.1). Normality was assessed with the Shapiro–Wilk test at an alpha level of 0.05. Parametric data were compared with the unpaired Student’s *t*-test, and nonparametric data were compared with the Mann-Whitney test. The Kruskal–Wallis test and post hoc Dunn’s multiple comparisons test were used for subgroup analysis. Statistical significance was defined as *p* < 0.05. No a priori sample size calculation was performed for this exploratory, retrospective study.

## Results

### Cohort characteristics

In total, 140 cases were analyzed. Of these, 22 patients (15.7%) presented with a malignant tumor comprising 10 (45.5%) patients with metastasis of a squamous cell carcinoma, six (27.7%) patients suffering from lymphoma, three (13.6%) with adenocarcinoma, two (9.1%) with mucoepidermoid carcinoma, and one (4.6%) with carcinoma ex pleomorphic adenoma, respectively. The remaining 118 tumors were benign comprising 60 (50.8%) Warthin’s tumors, 33 (28.0%) pleomorphic adenomas, six (5.1%) oncocytomas, four (3.4%) lymphoepithelial cysts, and three (2.5%) basal cell adenomas, respectively. There were two cases each (1.7%) of cyst, lipoma and lymph node, and one case (0.8%) each of lymphoepithelial lesion, nodular fasciitis, sarcoidosis, myoepithelioma, schwannoma, and cystadenoma. The mean patient age was 60.1 ± 14.5 years. There were 81 male patients (57.9%) and 59 female patients (42.1%).

### Taller-than-wide and risk of malignancy

**Fig. 2:**
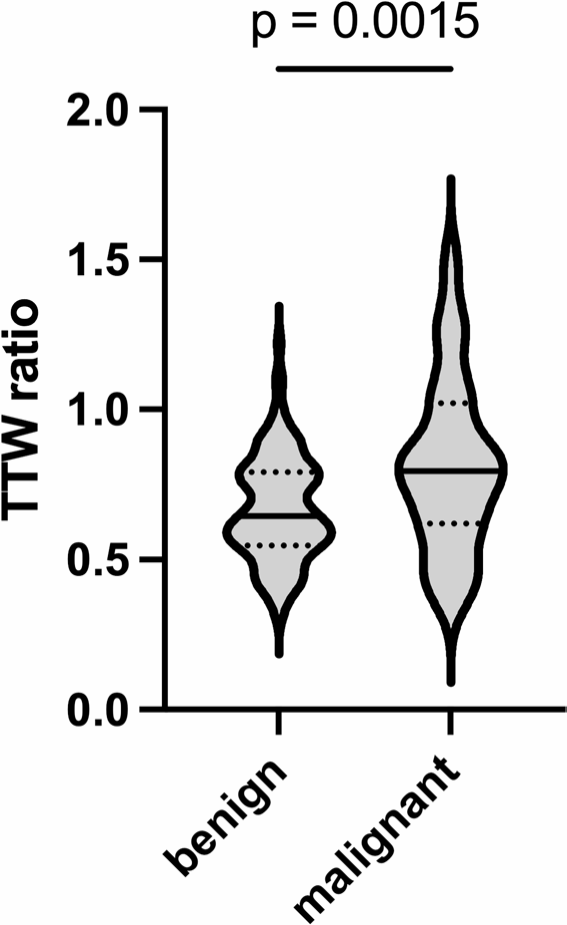
Violin plot showing the median (solid line) and quartiles (dotted lines) of the TTW ratio for benign and malignant parotid gland tumors, as confirmed by histopathology

Among the malignant tumors, six (27.3%) were taller-than-wide, while 16 (72.7%) were wider-than-tall. This proportion was significantly higher than in the benign group, where only four (3.4%) were taller-than-wide and 114 (96.6%) were wider-than-tall (*p* < 0.01). The mean TTW ratio was also higher in the malignant group (0.81 ± 0.29) than in the benign group (0.67 ± 0.17, *p* < 0.01, Fig. 2).

**Fig. 3:**
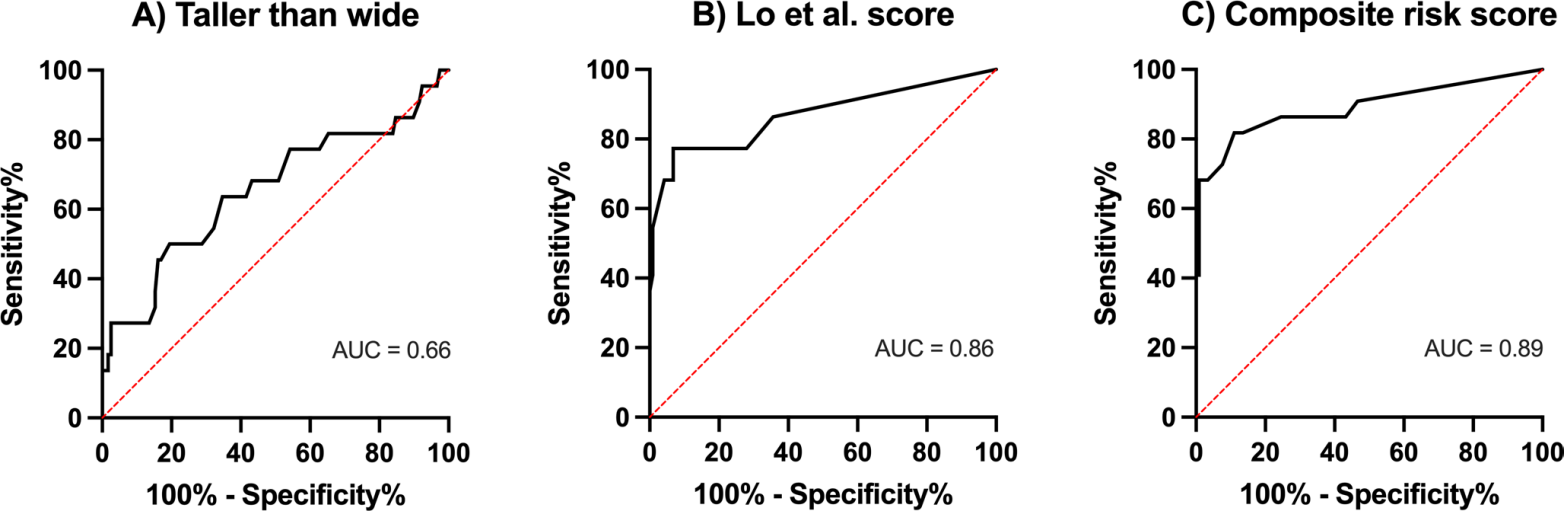
Receiver operating characteristic (ROC) curves for (**A**) the taller-than-wide (TTW) ratio alone, (**B**) the score proposed by Lo et al., and (**C**) the composite risk score augmenting the score with the TTW ratio

Receiver operating characteristic (ROC) curve analysis was used to determine the TTW ratio’s ability to distinguish between benign and malignant parotid tumors. The area under the curve (AUC) was 0.66 (Fig. 3 A). The highest Youden’s index (sensitivity + specificity − 1) was achieved with a TTW ratio cutoff of 0.82, yielding a sensitivity of 50.0% and a specificity of 80.5%. The cutoff ratio of 1 was also evaluated—corresponding to a visually apparent taller-than-wide shape and eliminating the need for numerical calculation—and resulted in a specificity of 96.6% and a sensitivity of 27.3%.

### Other factors associated with malignancy

Malignant tumors were more frequently corresponding to a lower long-to-short axis ratio (1.51 ± 0.46 vs. 1.65 ± 0.43, *p* < 0.05). Patients with malignant tumors were more likely to have lymph node involvement outside the parotid gland (27.3% vs. 0%, *p* < 0.0001), or present with facial nerve paralysis (22.7% vs. 0%, *p* < 0.0001). Their mean age was higher (68.0 ± 15.2 vs. 58.6 ± 13.9 years, *p* < 0.01), they were more likely to be non-smokers (90.9% vs. 44.1%, *p* < 0.0001), and they had a higher incidence of head and neck skin malignancy in their history (36.4% vs. 0.9%, *p* < 0.0001). Malignant tumors more often had irregular borders (50.0% vs. 3.4%, *p* < 0.0001), showed skin infiltration (27.3% vs. 0%, *p* < 0.0001), calcification (18.2% vs. 0%, *p* < 0.001), had a non-homogenous appearance (81.8% vs. 56.8%, *p* < 0.05), and were less likely to be avascular on color Doppler sonography (15.0% vs. 39.0%, *p* < 0.05).

There were no significant differences between the malignant and benign groups in terms of sex, pain, B symptoms, shape, or maximum tumor size (all *p* > 0.05). The clinical characteristics and ultrasound criteria are summarized in Tables 1 and 2, respectively.

**Table 1:** Clinical characteristics of patients presenting with benign or malignant tumor in final histology

Clinical characteristic	Benign (*n* = 118)	Malignant (*n* = 22)	*p* value
Age, mean ± SD, years	58.6 ± 13.9	68.0 ± 15.2	**0.0055**
Sex, female, No. (%)	52 (44.1)	7 (31.8)	0.3509
Facial nerve paralysis, No. (%)	0 (0)	5 (22.7)	**< 0.0001**
Pain, No. (%)	14 (11.9)	5 (22.7)	0.1819
B symptoms, No. (%)	2 (1.7)	0 (0)	> 0.9999
History of head and neck skin cancer, No. (%)	1 (0.9)	8 (36.4)	**< 0.0001**
Active smoking, No. (%)	66 (55.9)	2 (9.1)	**< 0.0001**

**Table 2:** Ultrasound criteria for tumors defined as benign or malignant in final histology. *Color doppler sonography to assess vascularity was performed in 120 patients in total: 100 with benign tumors and 20 with malignant tumors

Ultrasound criterion	Benign (*n* = 118)	Malignant (*n* = 22)	*p* value
Maximum tumor size, mean ± SD, mm	23.0 ± 7.5	21.7 ± 7.9	0.4713
Long-to-short axis ratio, mean ± SD	1.65 ± 0.43	1.51 ± 0.46	**0.0301**
Taller-than-wide ratio, mean ± SD	0.67 ± 0.17	0.81 ± 0.29	**0.0015**
Taller-than-wide criterion, No. (%)	4 (3.4)	6 (27.3)	**0.0011**
Multiple tumors, ipsi- or contralateral, No. (%)	27 (22.9)	4 (18.2)	0.7839
Contralateral tumor present, No. (%)	16 (13.6)	1 (4.5)	0.4724
Irregular borders, No. (%)	4 (3.4)	11 (50.0)	**< 0.0001**
Infiltration of skin, No. (%)	0 (0)	6 (27.3)	**< 0.0001**
Non-homogenous appearance, No. (%)	67 (56.8)	18 (81.8)	**0.0325**
Avascular in color Doppler sonography*, No. (%)	39 (39.0)	3 (15.0)	**0.0433**
Extraglandular lymph node involvement, No. (%)	0 (0)	6 (27.3)	**< 0.0001**
Calcification, No. (%)	0 (0)	4 (18.2)	**0.0005**

### Composite risk score calculation

Application of the score proposed by Lo et al. and Cheng et al. [9, 13] yielded a significantly higher score in malignant than in benign tumors (3.43 vs. 0.57, *p* < 0.0001). ROC curve analysis resulted in an AUC of 0.86 with an ideal cutoff determined by Youden’s index of 1.6 and above, resulting in a sensitivity of 77.3% and a specificity of 93.2% (Fig. 3B).

The score was augmented with information about the TTW ratio. A dichotomized TTW-ratio variable was introduced, defined as positive if the TTW ratio was ≥ 0.82, as determined by the highest Youden’s index. The best AUC was achieved by adding a weighting factor of 1.5, resulting in the following formula: 2.08 × (boundary) + 1.75 × (regional lymphadenopathy) + 1.18 × (shape) + 1.45 × (posterior acoustic enhancement) + 2.4 × (calcification) + 1.5 × (TTW ratio). The composite score was significantly higher in malignant than in benign tumors (4.11 vs. 0.84, *p* < 0.0001) and yielded an AUC of 0.89, with a cutoff of 2.4 providing a sensitivity of 81.2% and a specificity of 89.0% (Fig. 3 C).

### Subgroup analysis

A subgroup analysis compared benign tumors (*n* = 118) with carcinoma (*n* = 16) and lymphoma (*n* = 6). The Kruskal–Wallis test revealed significant differences between groups for both the TTW ratio (*p* < 0.01) and the composite risk score (*p* < 0.0001). Post hoc Dunn’s multiple comparisons test showed significant differences when comparing benign vs. carcinoma both for TTW ratio (*p* < 0.01, supplementary Fig. 1) and the composite risk score (*p* < 0.0001), but no significant difference when comparing benign vs. lymphoma or lymphoma vs. carcinoma (all *p* > 0.05).

## Discussion

This study investigated the diagnostic value of TTW on ultrasound for differentiating benign from malignant parotid gland tumors. Malignant tumors were significantly more likely to display a TTW shape than benign tumors. This observation aligns with findings in other anatomical locations, such as the thyroid and breast, where TTW is established as a malignancy marker, reflecting the diminished respect for microanatomic boundaries of malignant cells [19, 20, 24].

Despite the promising specificity (96.6%) for a TTW ratio > 1, the sensitivity was low (27.3%). A substantial subset of cancers in our series still exhibited the more common wider-than-tall shape. Therefore, TTW should be considered as an adjunctive red flag rather than a standalone diagnostic criterion.

Lo et al. developed a five-parameter model for predicting malignancy in salivary gland tumors including unclear boundary, regional lymphadenopathy, irregular or spiculated shape, absence of posterior acoustic enhancement, and presence of calcification, with each feature weighted according to logistic regression coefficients. Using a cutoff score of ≥ 3, their model achieved an AUC of 0.90, with 70.2% sensitivity, and 93.9% specificity in the original cohort [9]. The score was validated in a separate cohort at the same institution with a reported AUC of 0.82, 58% sensitivity, and 89% specificity [13]. In our cohort, the score resulted in an AUC of 0.86, indicating good discriminatory ability, thus achieving external validation of the score for the first time. The score was then augmented with the TTW ratio. This resulted in an AUC of 0.89, further improving the discriminatory ability of the score.

Both using TTW as a red flag and applying the composite risk score could help guide surgical planning, e.g., identifying those patients with high risk of malignancy, who should undergo fine-needle aspiration or core needle biopsy prior to surgical resection [17, 25], which in turn leads to higher rates of clear pathologic margins and upfront neck dissection [26, 27]. Neither TTW nor the proposed risk score can substitute for histological sampling, which remains indispensable for definitive diagnosis.

Evaluation of TTW does not require additional equipment or extensive training, providing a low-cost and widely accessible metric that can be easily incorporated into the risk score. This stands in contrast to advanced ultrasound techniques that have been evaluated for parotid gland tumors. Contrast-enhanced ultrasound (CEUS) has demonstrated value in assessing tumor vascularization and perfusion dynamics in salivary gland lesions [28]. Differences in peak intensity and time-to-peak enhancement between benign and malignant tumors have been reported [8] and distinct microvascularization patterns among pleomorphic adenomas, Warthin tumors, and malignant lesions have been described [12]. Elastography, specifically shear-wave elastography (SWE) and acoustic radiation force impulse (ARFI) imaging, has also been evaluated for differentiating benign from malignant lesions based on tissue stiffness [29–31]. Significantly higher stiffness values in malignant tumors using Virtual Touch Quantification (VTQ) and Imaging Quantification (VTIQ) have been found [10] and higher strain scores in ARFI could predict malignancy with a specificity of 81% and a sensitivity of 63% [32]. Neither CEUS nor elastography is standard of care yet.

This study has several limitations. First, its retrospective, single-center design inherently limits the generalizability of the findings. Second, a substantial proportion of malignant tumors in our cohort were metastases from cutaneous squamous cell carcinomas rather than primary parotid malignancies. While subgroup analysis revealed significant differences between carcinomas and benign tumors, the sample size was insufficient for further evaluation of lymphomas or other subgroups. Therefore, the applicability of our results to primary parotid gland carcinomas may be restricted. Third, while the TTW parameter is theoretically objective, it may be subject to variability based on transducer pressure, angulation, and patient positioning. Though interrater reliability was not assessed in this study due to the retrospective design, the literature from thyroid and breast ultrasound indicates that assessment of tumor shape, including TTW, generally shows moderate to substantial interrater reliability (κ ≈ 0.5–0.7), and is considerably more reproducible than other sonographic features such as margins or echogenic foci [33–36].

Future studies need to validate the TTW criterion and the composite risk score in larger, prospective multicenter cohorts, which would allow a more representative spectrum of salivary gland pathologies and provide robust estimates of diagnostic accuracy across different practice settings. In addition, interobserver reliability should be systematically assessed, and subgroup-specific analyses—such as differentiation among histological subtypes of malignancy—should be pursued. Looking forward, with the increasing use of AI in ultrasound image analysis, TTW should be incorporated into machine learning algorithms as an automatically extracted parameter, potentially improving diagnostic accuracy, speed, and reproducibility [23, 37, 38]. Such approaches may also mitigate human interobserver variability and support more standardized reporting across institutions. Ultimately, the combination of prospective multicenter validation and incorporation into AI-driven diagnostic models will be essential to establish the clinical utility of TTW in routine practice.

In conclusion, the TTW ratio represents a simple yet highly specific sonographic parameter for the identification of malignant parotid gland tumors. Although it lacks sufficient sensitivity for standalone use, its incorporation into multiparametric or algorithmic-driven diagnostic models could enhance preoperative risk stratification and guide surgical planning.

## Supplementary Information

Below is the link to the electronic supplementary material.

Supplementary figure 1 (PNG 64.1 KB)

High Resolution Image Fig. 1: Scatter plot of the subgroup analysis comparing benign, lymphoma and carcinoma patients including p-values from the post hoc Dunn’s multiple comparisons test.(TIF 131 KB)

## Data Availability

The datasets are not publicly available due to patient privacy restrictions but are available from the corresponding author on reasonable request, subject to institutional and GDPR requirements.
