# Supplementary figures and images for: Taller-than-wide as a red flag for malignancy in ultrasound of parotid gland tumors
Source: Eur Arch Otorhinolaryngol. 2025 Nov 12;283(1):449–55. doi: 10.1007/s00405-025-09832-9 (PMC12904917; doi:10.1007/s00405-025-09832-9)

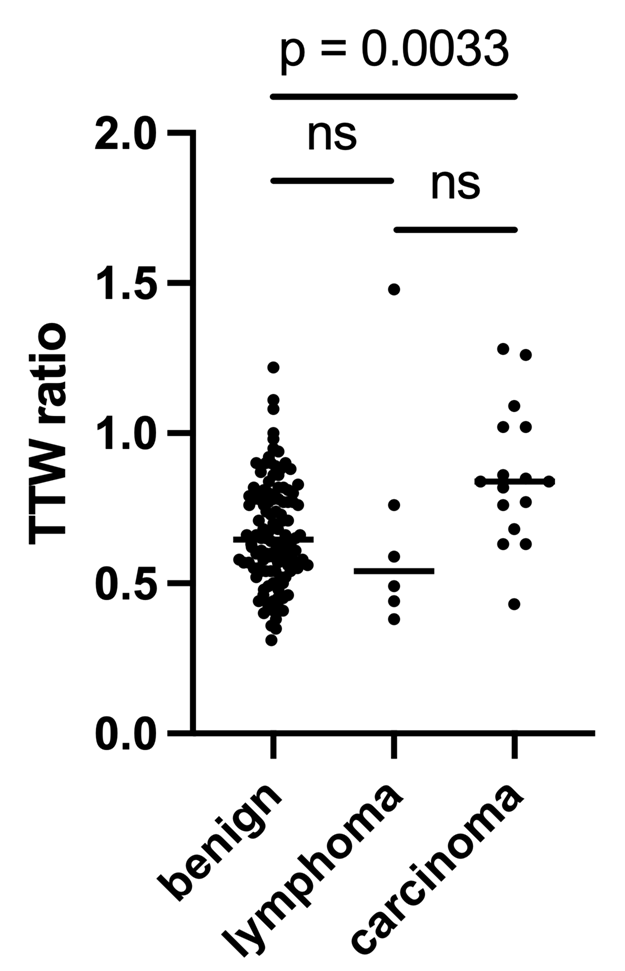

Supplement: Supplementary file 1 — (PNG 64.1 KB) [file 405_2025_9832_Fig4_ESM.png]

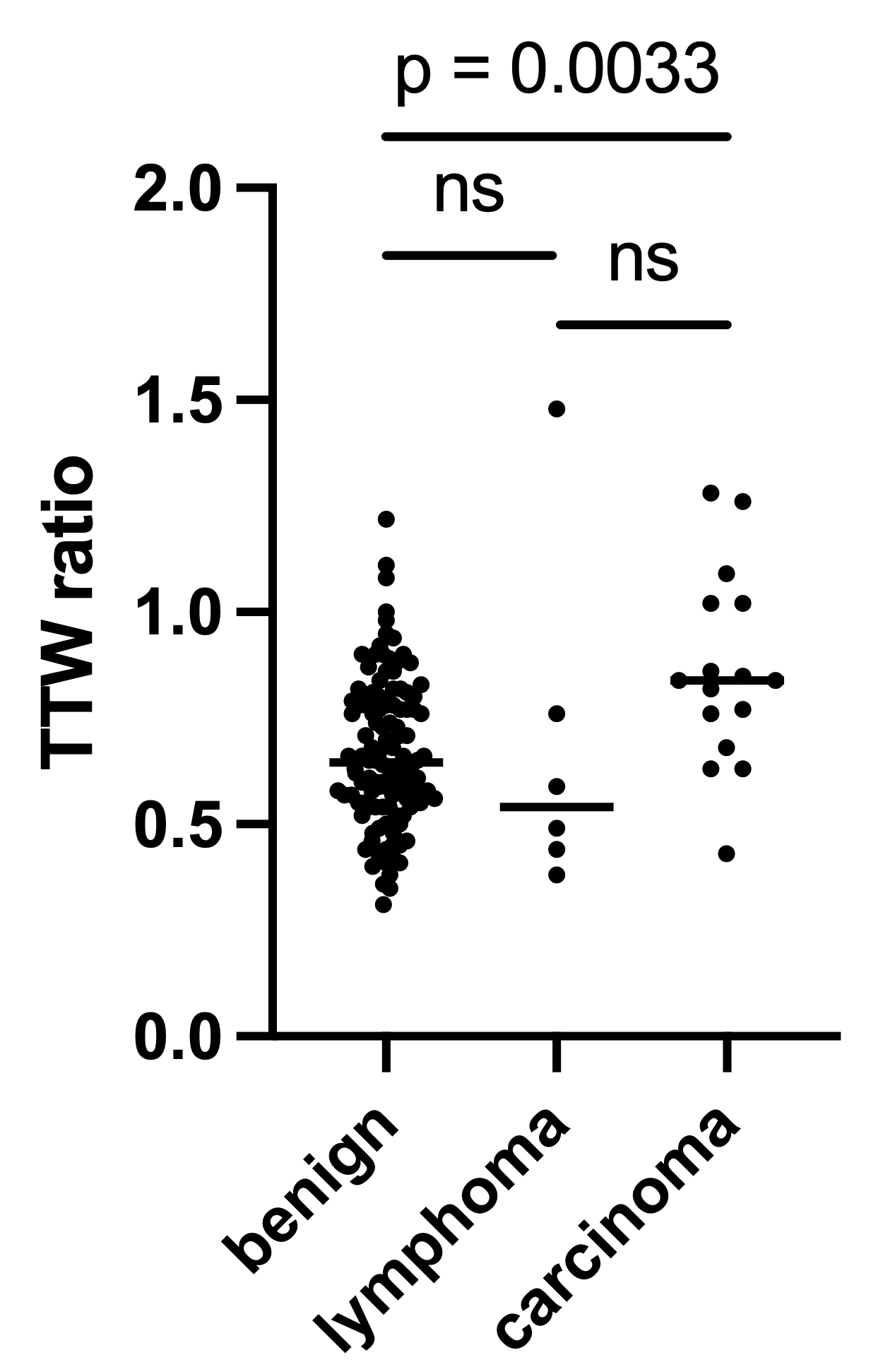

Supplement: Supplementary file 2 — High Resolution Image Fig. 1: Scatter plot of the subgroup analysis comparing benign, lymphoma and carcinoma patients including p-values from the post hoc Dunn’s multiple comparisons test.(TIF 131 KB) [file 405_2025_9832_MOESM1_ESM.tiff]
